# Literary Notes

**Published:** 1900-01

**Authors:** 


### LITERARY NOTES.

THE Antikamnia Chemical Company, of St. Louis, has distributed to its patrons another skeleton calendar, the drawings of which were made by that clever artist, the late Dr. L. Crusius. This calendar for 1900 is, we think, superior in design and effect to its predecessors. The figures are for the most part smaller, and the subjects are better in conception and execution. It will be sent to physicians upon application to the Antikamnia Chemical Co., 1723 Olive Street, St. Louis, Mo.

THE Columbia desk calendar for 1900 has come to hand with its usual promptitude. This is its fifteenth edition and like its predecessors, this calendar is designed to furnish the user with a convenient memorandum pad arranged according to the days of the year; and, incidentally, to supply valuable guidance to all seekers for information on the subject of quality in bicycles. It is needless to add that it fulfils both conditions admirably. It has become well-nigh an indispensable part of the busy man's desk outfit.

---

ARCHIVES OF NEUROLOGY AND PSYCHOPATHOLOGY.—The Archives of Neurology and Psychopathology, published under the auspices of the New York State Hospitals and the Pathological Institutes commences its second year under the most auspicious circumstances. The opening number is a handsome volume of extreme value and importance and is comparable with the best of medical publications anywhere to be found.

The contents of this number are: On the Nucleoproteids of the brain, by P. A. Levene. Introduction. Some remarks on the chemical nature of the chromatins and their rôle in the function of the cell. Cerebro-nucleoproteid. Methods of preparation. Composition. Cerebronuclein. Cerebronucleic acid. Some remarks in regard to the presence of more than one chromatin in the nerve cell.

Iodine compounds in the tissues after administration of potassium iodide, by P. A. Levene. Introduction. The purpose of the investigation. Methods employed. Results of the analysis of the egg. Results of the analysis of other tissues.

The cranial and first spinal nerves of Menidia, by C. J. Herrick. A contribution on the nerve components of the bony fishes.

The article or rather monograph by Dr. Herrick is one of the most important ever made to ichthyology and is accompanied by seven handsome plates.

The editors are to be congratulated upon the great success of their undertaking and the profession should encourage in all manner possible the efforts of the distinguished staff of editors.

---

MESSRS. M. J. BREITENBACH COMPANY, of 100 Warren Street, New York, have again placed the medical profession under great obligations. The edition of their year-book for the year 1900 has been sent out to physicians and is now being distributed through the mails. It is a handsome duodecimo volume, bound in flexible red muslin, containing a page for every day in the year. Its frontispiece is a fine portrait of the late Lawson Tait. It is one of the most convenient desk memorandum books that we have seen and has become almost indispensable for an active physician. It will be supplied to those who have, perchance, been overlooked in the first distribution, upon application to the well-known house that publishes it.

---

Messrs. Battle & Co., of St. Louis, have recently issued a brochure, entitled, Blood dyscrasia, in which the value of ectol as an "anti-purulent" is set forth. The testimony as to the value of the remedy is quite voluminous and would seem to justify further experimentation with it in cases with a tendency to dyscrasia, tissue disintegration and blood poisoning.

---

Coca and its therapeutic application is the title of an interesting and instructive monograph written by Mr. A. Mariani, of Paris, which is well illustrated. It is based on physiological studies, researches and designs made at Mr. M.'s laboratory at Neuilly on the Seine, where in hot houses he has succeeded in producing large quantities of coca plants, that are now under cultivation. Physicians are invited to visit this establishment and no doubt many will avail themselves of the invitation during the Paris exposition.

Erythroxylon coca is a drug of great therapeutic value, yet it is greatly misunderstood and even misrepresented. The work Mr. Mariani is doing will serve to clear up doubts and place it where it properly belongs, as he is undoubtedly the greatest living expert upon this plant and its products. The book in question may be obtained upon application to Mariani & Co., 52 West 15th St., New York.

